# Materials design for thermally improved safety in lithium-ion batteries

**DOI:** 10.1039/d5sc08060f

**Published:** 2026-01-14

**Authors:** Songpei Nan, Guoxin Gao, Wei Yu, Shujiang Ding, Dawei Ding

**Affiliations:** a School of Chemistry, Xi'an Jiaotong University, Engineering Research Center of Energy Storage Materials and Devices, Ministry of Education Xi'an 710049 China dingsj@mail.xjtu.edu.cn davidding1@mail.xjtu.edu.cn

## Abstract

With the ever-increasing demand for high-energy-density lithium-ion batteries (LIBs) in multiscale energy storage, safety concerns have emerged as critical obstacles hindering their widespread application. The excess heat generated during the electrochemical process, if not properly managed, can accumulate and accelerate the aging of key cell components, potentially leading to catastrophic thermal runaway events such as fires and explosions. Thus far, considerable attention has been devoted to alleviating intense thermal runaway through fire-safe materials and energy-intensive thermal management technologies. However, the stabilization of the electrochemical environment through intrinsic thermal dissipation and temperature regulation governed by key material design has received comparatively little consideration. This paper aims to summarize the mechanism of thermal runway and highlight material advances for safer LIBs, with particular emphasis on the thermal-electrochemical synergy in mitigating localized overheating, stabilizing the electrochemical environment, and improving electrochemical performance. Subsequently, recent research progress in thermal management materials and strategies for dynamic temperature regulation is reviewed. Finally, current challenges are discussed, and future directions are proposed for material innovations that can be applied to high-energy-density and high-safety LIBs.

## Abstract 2

Material advances in key cell components and external thermal management for improving lithium-ion battery safety.

## Graphical abstract

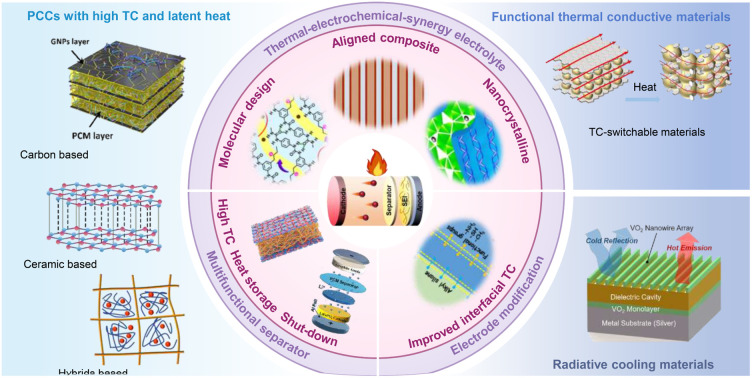

## Introduction

The transition from fossil fuels to renewable energy-powered industry, transportation, and human activities is widely regarded as the ultimate solution to environmental challenges caused by fossil fuel combustion. Energy storage devices play an irreplaceable role in grid-connected renewable energy systems and electric vehicle applications. Among rechargeable energy storage technologies, lithium-ion batteries (LIBs) have emerged as the predominant choice due to their high energy density, long cycle life, and the absence of memory effects. Nevertheless, safety concerns surrounding LIBs have persisted since their commercialization, primarily arising from thermal runaway-induced battery fires.^1,2^

**Fig. 1:**
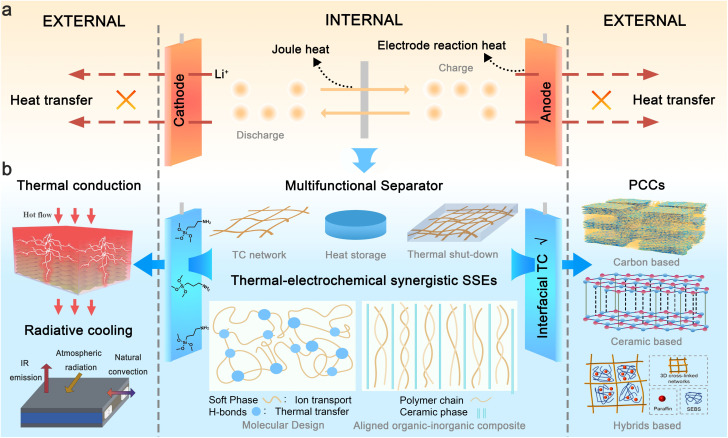
(a) Diagram of heat generation and accumulation during LIB operation, (b) material advances in key cell components and external thermal management for improving battery safety. Reproduced from ref. 9, copyright 2022, Elsevier. Reproduced from ref. 10, copyright 2019, Elsevier. Reproduced from ref. 11, copyright 2023, Elsevier.

Thermal runaway progression in LIBs typically originates from heat accumulation dynamics, governed by the imbalance between heat generation and dissipation.^3^ Commercial LIBs operate through the shuttling of Li-ions in liquid electrolytes between cathodes and anodes (Fig. 1a), generating heat from joule heating and electrode reaction. Under low charging/discharging rates (<1C), generated heat can be dissipated spontaneously. However, during fast-charging (>3C) or high-power discharging scenarios (common in electric vehicle application), excessive heat generation leads to significant heat accumulation.^4,5^ Furthermore, anisotropic thermal conductivity (TC) within cell components and non-uniform cooling conditions induce localized temperature gradients.^6^ These hotspots accelerate electrochemical reaction kinetics, creating a positive feedback loop: elevated temperatures increase local current density, which further exacerbates heat generation. When cell temperature exceeds abuse thresholds, thermal runaway—characterized by uncontrolled heat release due to exothermic chemical chain reactions—is triggered, culminating in catastrophic fires or explosions.^1^ External abuse conditions such as overheating, overcharging, and mechanical abuse can also initiate thermal runaway through distinct failure pathways.^7,8^

Substantial efforts to enhance battery safety over recent decades have focused on materials advances and thermal management systems. Materials advances mainly focus on safe electrolytes,^12–15^ separator engineering, thermally stable electrodes,^16^ and smart functional materials. Safe electrolytes include non-flammable electrolytes,^17^ flame retardant additives,^18^ and stable lithium salts.^19^ However, these attempts often introduce new challenges. For example, directly adding flame retardants to the electrolyte often compromises the battery's electrochemical performance.^20^ Recent studies demonstrate that decoupling ion transport with flame-retardant properties through the introduction of a triadic molecular synergy system can mitigate this adverse effect.^21^ Solid-state electrolytes with advanced safety features are considered promising candidates for next-generation batteries but may still exhibit heat generation comparable to carbonate-based electrolytes when paired with electrode materials and can ignite once temperatures reach a certain threshold.^22,23^ Separator thermal stability can be improved through coating polymers with inorganic materials (*e.g.*, SiO_2_, Al_2_O_3_, TiO_2_, and ZrO_2_) or using materials of high melting temperatures with low shrinkage at elevated temperatures, such as polyimide, cellulose, and polyesters.^24–26^ Modifications of electrode materials through doping,^27^ surface coating,^28^ or an artificial cathode-electrolyte interphase/solid electrolyte interphase (SEI)^29^ are common strategies to enhance thermal stability, albeit often at the expense of energy density.^30^ Recently, an emerging class of safe functional materials (*e.g.*, shutdown materials) has been developed to protect batteries from short-circuit-induced heat generation without side effects under normal operating conditions. These materials can be integrated into existing battery structures, such as the current collector,^31^ electrolyte,^32–34^ separator,^35^ or electrode.^36^ These shutdown materials, primarily composed of thermosensitive polymers, operate by sharply increasing internal cell resistance at high temperatures through thermal expansion or melting, thereby blocking electron conduction or Li^+^ pathways.^37,38^ Most shutdown materials primarily focus on mitigating late-stage thermal runaway phenomena, often at the expense of the battery's electrochemical performance or by relying on irreversible safety mechanisms that lead to permanent battery failure.^39–42^ In this regard, the introduction of reversible thermal shutdown materials is more advantageous for the recovery of electrochemical performance.^43,44^ In addition to the shutdown mechanism, slowing the crosstalk diffusion kinetics of lithium ions and active gases through the temperature-triggered formation of a dense crosslinked polymer network has been identified as an effective strategy to prevent the escalation of thermal runaway.^45^

Thermal management systems primarily involve the implementation of active cooling strategies (*e.g.*, forced air/liquid convection,^46^ thermoelectric devices^47^) and passive thermal regulation materials (*e.g.*, phase changing materials (PCMs),^48^ heat pipes,^49^ radiative cooling materials^50^). While active systems demonstrate superior cooling capacity and are particularly effective for addressing specific thermal abuse scenarios, their enormous parasitic energy consumption limits their application.^51–53^ Besides, conventional thermal management systems are typically applied externally to dissipate heat from the battery surface. However, this approach can induce undesired internal temperature elevation and thermal gradient due to inefficient heat transfer from the core to surface.^54,55^ In contrast, internal thermal management through material innovation offers enhanced safety and durability by directly regulating temperature distribution within the cell. Nevertheless, research in this aera remains in its early stage, necessitating further efforts to advance these technologies for practical implementation.

Although recent reviews have highlighted advancements in thermal management to enhance the safety of LIBs, most have primarily focused on the exploration of thermally stable battery components^56–58^ or external cooling technologies.^59–61^ There is a critical need to fundamentally prevent thermal runaway at its initial heat accumulation stage through a comprehensive design of key materials, both internally and externally. This paper aims to systematically examine advanced thermal management strategies and propose material design directions for improving the electrochemical environment to prevent thermal runaway initiation from a holistic perspective, with an emphasis on the thermal-electrochemical synergy that can significantly enhance both electrochemical performance and battery safety (Fig. 1b). Specifically, enhancing thermal homogeneity within the battery can stabilize the electrochemical environment. As a result, more stable and uniform ionic transportation and deposition can prevent hotspot generation and lithium dendrite formation. In the material design of key battery components, such as polymer-based solid electrolytes, we particularly focus on reconciling the trade-off between the thermal and electrochemical properties through materials engineering. Besides, recent research progress in thermal management materials and strategies for dynamic temperature regulation is reviewed. Finally, we summarize contemporary challenges in battery thermal management materials and propose a roadmap for safety design methodologies in next-generation LIBs.

### Mechanism for thermal runaway

**Fig. 2:**
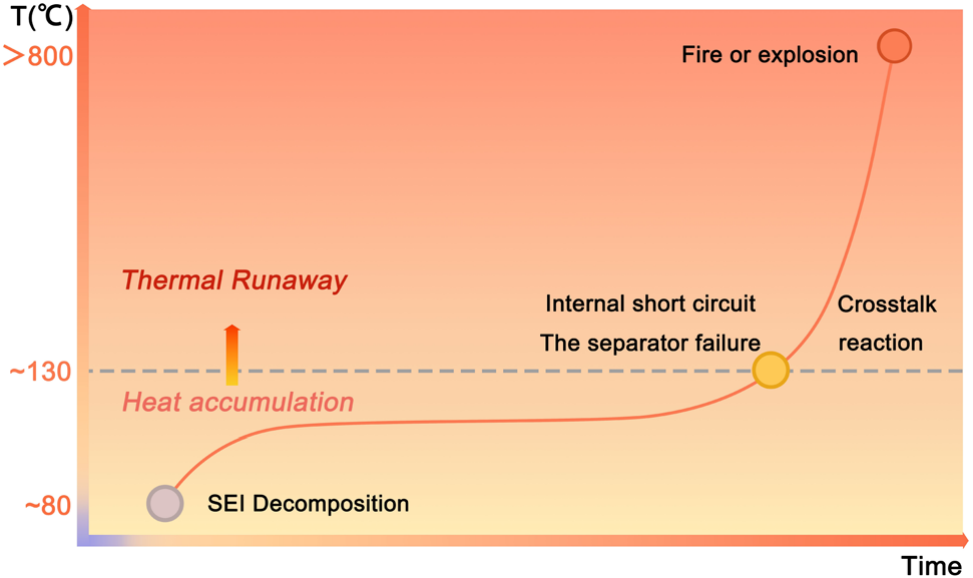
Thermal runaway process of LIBs.

A deep understanding of the thermal runway mechanism is critical for analyzing challenges and formulating mitigation strategies. Although definitions of the thermal runaway stages vary across studies, the process can generally be divided into two stages: heat accumulation and intense thermal runaway (Fig. 2).^1,62–64^ The heat accumulation stage begins with the decomposition of the SEI layer on the anode surface and subsequent anode-electrolyte reactions at 80–110 °C. The relatively low cell temperature results in slow exothermic reaction rates, yielding mild heat release (∼257 J g^−1^). Consequently, the cell temperature rises at a moderate rate (<0.05 °C min^−1^), leading to a prolonged duration (minutes to days) of the heat accumulation stage.^65,66^

If the generated heat cannot be dissipated, continuous heat accumulation may trigger the intense thermal runaway stage (130–300 °C), characterized by chemical chain reactions or crosstalk reactions.^67,68^ Polyolefin-based separators melt at ∼130 °C, causing internal short circuits and localized current surges.^69^ At temperatures above 200 °C, the cathode materials such as high-Li layered oxides decompose, releasing O_2_. The liberated O_2_ will react exothermically with carbonate solvents (*e.g.*, ethylene carbonate, which autoignites at 140 °C in air), generating CO and CO_2_, and releasing intense heat (>2000 J g^−1^).^36,70–72^ Finally, the intense chemical reaction kinetics accelerate exponentially, driving temperatures to over 800 °C within seconds (heating rate >104 °C min^−1^), ultimately causing uncontrollable fires or explosions.^73–75^

In the early stage of heat accumulation, enhancing internal heat dissipation through key cell components, such as separators or electrolytes with high TC and latent heat, which are capable of buffering sharp temperature variations, can effectively mitigate the escalation of thermal runaway. However, once the temperature surpasses a critical threshold during the intense thermal runaway stage, it necessitates the use of more thermally stable components or shutdown materials to suppress chain reactions or chemical crosstalk. Thus, it is more rational to address and curb thermal runaway during the early heat accumulation stage. As previously stated, peak internal temperatures during thermal runaway can exceed 800 °C. While introducing nonflammable additives could theoretically enhance safety, these materials must satisfy two stringent criteria: chemical stability when paired with all battery components and minimal impact on electrochemical performance. However, such requirements severely restrict viable options. Furthermore, dissipating heat during intense thermal runaway is practically infeasible due to the enormous cooling power required.^2^ Therefore, this study focuses on the early-stage thermal management through rationally designed materials that could prevent heat accumulation. In Section 3, we systematically analyze emerging thermal management strategies, including internal heat dissipation, external thermal regulation, and design principles. These insights aim to guide the development of next-generation safe LIBs with intrinsic thermal management.

## Battery materials design

### Internal heat dissipation

Heat accumulation within a battery can lead to uneven temperature distribution and temperature rise if the heat is not dissipated promptly. This can accelerate battery aging, cause component failure, and ultimately trigger thermal runaway if the temperature exceeds a critical threshold.^76^ Therefore, internal thermal management must address heat accumulation and temperature non-uniformity by enhancing heat dissipation within the battery. This relies on the optimal design of key cell components, including separators,^77^ electrolytes,^78^ and electrodes.^79^

#### Separator

In conventional LIBs with liquid electrolytes, the separator plays a critical role in thermal regulation and internal heat dissipation. Common strategies focus on improving mechanical strength and thermal stability by incorporating inorganic fillers into polymer-based separators.^80^ These modifications aim to suppress lithium dendrite growth and raise the polymer's melting point. However, less attention has been paid to mitigating local overheating through the use of high-TC separators.

Studies have shown that the application of high-TC ceramic coating materials not only enhances the thermal stability of polymer separators but also significantly reduces temperature rise during high-rate discharge, thereby improving electrochemical performance.^81^ Furthermore, the combination of highly heat-resistant polymer matrix materials (*e.g.*, poly-*p*-phenylene terephthamide, polyimide) with thermally conductive fillers (*e.g.*, boron nitride (BN), carbon nanotubes) optimizes electrochemical performance through synergistic mechanical-thermal-electrochemical effects.^82–85^ For instance, Lim *et al.* developed a highly thermally conductive composite separator composed of interwoven super-aligned carbon nanotubes and super-aligned BN@carbon nanotubes.^86^ This separator features a uniform thermal field, enabling rapid heat dissipation to prevent overheating while suppressing the polysulfide shuttle effect and lithium dendrite growth, thereby enhancing electrochemical performance.

**Fig. 3:**
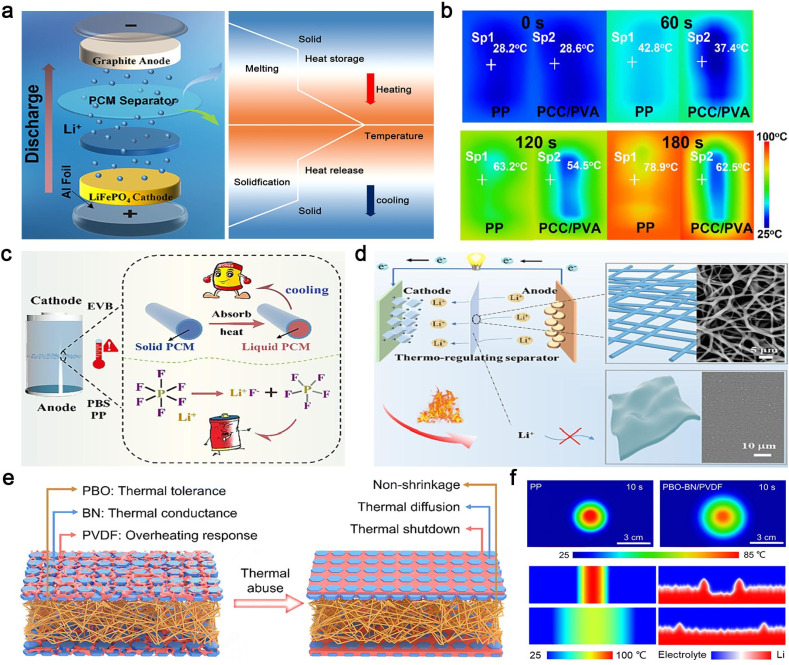
Separator materials for LIBs. (a) Battery structure and discharge diagram, (b) thermal management images of the PP separator and phase change capsules/poly(vinyl alcohol) (PCC/PVA) separator heated at 100 °C. Reproduced from ref. 87, copyright 2024, American Chemical Society. (c) High-performance LIB based on a PEG/PVDF@PBS separator, (d) battery thermal management based on the PEG/PVDF@PBS separator. Reproduced from ref. 90, copyright 2024, Elsevier. (e) Design illustration of the thermal overheating-responsive separator, (f) thermal infrared images (top-down view) of the PP and PBO–BN/PVDF separators, and the simulated Li deposition morphology on the Li metal surface (cross-sectional view) under different temperature distributions. Reproduced from ref. 91, copyright 2024, The Royal Society of Chemistry.

Recently, PCMs with high enthalpy and rapid temperature response have gained significant attention for their ability to endow separators with excellent heat dissipation capabilities. Liu *et al.* proposed a novel phase-change functional separator inspired by the structure of a sugar gourd.^87^ The separator, made of melamine-encapsulated paraffin, rapidly absorbs heat generated during battery operation. By mimicking the nutrient-water transport mechanism of natural gourds, it significantly enhances the melting and heat storage efficiency of the PCM (Fig. 3a). Experimental results demonstrate that this bionic structure not only effectively slows the rate of temperature rise within the battery (Fig. 3b) but also enables rapid heat dissipation during short-circuit events, providing an innovative solution to prevent thermal runaway.

Previous studies have demonstrated that thermosensitive polymer separators/electrolytes can serve a dual shutdown function by suppressing ionic transport and preventing crosstalk diffusion of active gases between electrodes when the temperature exceeds a critical threshold, thereby mitigating the escalation of thermal runaway.^45,88,89^ However, under rapid local overheating conditions caused by internal short circuits, the shutdown function may fail if the separator shrinks or melts. Unlike traditional strategies that rely on ceramic/polymer composite materials with high melting temperatures to improve separator thermal stability and safety, Wang *et al.* developed a polyethylene glycol/polyvinylidene fluoride (PEG/PVDF) @polybutylene succinate (PBS) core–shell separator with dual cooling and thermal shutdown functions using a one-step coaxial electrostatic spinning method (Fig. 3c).^90^ The cooling function of PEG in the separator effectively prevents ion conductivity degradation during aging, thereby mitigating specific capacity reduction and polarization voltage increase compared to conventional polymer separators (*e.g.*, polypropylene (PP), polyethylene (PE), PBS). When the temperature reaches 110 °C, the PBS shell melts to block electrochemical reactions without thermal shrinkage, preventing further escalation of thermal runaway (Fig. 3d).

Guided by electro-chemo-thermal process modeling, Lu *et al.* proposed a separator design principle that integrates thermal tolerance, thermal conductance, and overheating response to enhance the thermal safety of high-energy-density batteries.^91^ They utilized a poly(*p*-phenylene benzobisoxazole) (PBO) membrane as the thermally tolerant matrix and composite coating layers of BN nanosheets and PVDF as the thermally conducting and overheating-responsive layers, forming a sandwich trilayer separator (denoted as PBO–BN/PVDF) (Fig. 3e). The PBO–BN/PVDF separator exhibits high mechanical strength and excellent thermal stability (almost zero shrinkage at 350 °C), preventing internal short circuits by suppressing separator shrinkage. Additionally, the thermally conducting network enhances heat dissipation, eliminating heat accumulation and large temperature gradients within the cell, which is beneficial for preventing lithium dendrite growth (Fig. 3f). Under harsh thermal conditions, the PVDF layer triggers an overheating-responsive shutdown to cut off ionic transport, ensuring safe battery operation and preventing catastrophic thermal runaway. These optimized approaches to separator design address critical challenges in battery thermal management, enhancing both performance and safety. It is worth noting that the design principles of the separator are partially applicable to the polymer-based solid electrolyte as well, since it necessitates electrolytes with high thermal conductivity, stability, and considerable mechanical strength for effective heat dissipation. Furthermore, special attention must be given to addressing ionic transport in the polymer electrolyte, which introduces significant complexity to material design. This will be discussed in detail in the following section.

#### Electrolyte

Solid-state LIBs utilize solid electrolytes instead of flammable organic liquid electrolytes and separators, making them a promising next-generation battery technology with high energy density and enhanced safety features.^92^ Conventional liquid electrolytes face safety challenges such as dendrite growth, leakage, and flammability.^93,94^ Solid polymer electrolytes offer a viable solution to these issues, providing advantages such as good processability, high flexibility, and lightweight properties.^95^ However, the low TC of polymer electrolytes limits their thermal response, posing safety risks when temperatures exceed the polymer's melting point.^96,97^ Recent studies have demonstrated that high-TC polymer electrolytes can be achieved through polymer structure design,^98^ incorporation of inorganic conductive fillers,^99^ and control of polymer crystallinity domains.

#### Polymer structure design

The molecular structure design of polymer electrolytes must balance TC and ionic conductivity. Unlike metals, heat dissipation in polymers predominantly relies on phonons, which are vibrations of the molecular structure. The presence of irregular structures, lattice defects, and grain boundaries can cause phonon scattering, resulting in lower TC. High molecular chain regularity^100^ and strong intermolecular interactions,^101–104^ such as hydrogen bonding and π–π stacking, facilitate phonon propagation along molecular chains, minimize phonon scattering, and thereby enhance the thermal conductivity of polymers. The introduction of rigid chain segments^105^ (*e.g.*, aromatic rings, conjugated structures) or highly crystalline^106,107^ units can significantly enhance polymer TC. For example, Gu *et al.* improved the intrinsic TC of polydimethylsiloxane (PDMS) by 180% through the ring-opening copolymerization of liquid crystal siloxanes and octamethylcyclotetrasiloxane, which increased graft density.^108^ Conversely, ionic conductivity in polymers relies on flexible chain segments^109^ (*e.g.*, ether bonds, ester bonds) and polar groups^110,111^ to facilitate lithium-ion migration.

**Fig. 4:**
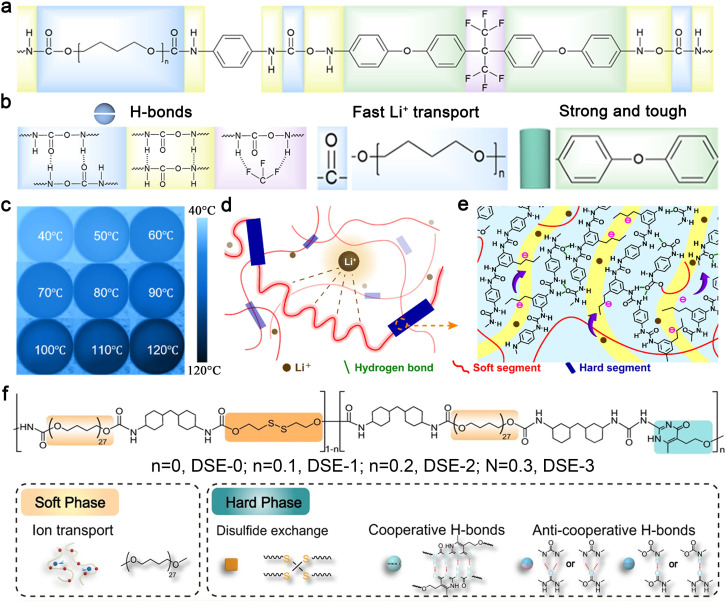
(a) Molecular formula of fluorine-containing supramolecular ionic conductive polyurethane elastomer (F-SMIU), (b) the role of each part of molecular design, (c) infrared thermogram of F-SMIU-9 (F-SMIU containing 9% filler) (40–120 °C). Reproduced from ref. 114, copyright 2025, Elsevier. Structural diagram of (d) a polyurethane-based anionic polymer with triflamide anions and (e) its hard phase. Reproduced from ref. 115, copyright 2023, American Chemical Society. (f) The chemical structure design and the proposed mechanism for the superior toughness of DSICE upon stretching. Reproduced from ref. 116, copyright 2022, Springer Nature.

The design of polymer electrolytes with dual thermal and ionic conductivity requires the incorporation of rigid chain segments and intermolecular hydrogen bonding to enhance phonon transport, as well as flexible chain segments to promote ionic migration. Additionally, mechanical strength is a critical consideration in polymer electrolyte design.^112^ Rigid segments (*e.g.*, aromatic rings) and multiple intermolecular hydrogen bonds^113^ can improve mechanical strength. Incorporating a supramolecular structure with a hard phase for phonon transport and mechanical strength, along with a soft phase for enhanced ionic dissociation and migration in the polymer electrolyte, can achieve thermal-electrochemical synergy, thereby enhancing the stability of the electrochemical environment and improving battery safety. Zhou *et al.* developed a quasi-solid-state polymer electrolyte using a dynamic supramolecular structure based on multiple dynamic bonds and a phase-locking strategy (Fig. 4a).^114^ As shown in Fig. 4b, C–F bonds and benzene rings in the hard phase enhance thermal stability, while C=O bonds and –CH_2_–O–CH_2_– groups in the soft phase facilitate Li^+^ coupling and migration, ensuring fast and uniform ion transport. Hydrogen bonds between hard phases endow the system with self-healing ability, high tensile strength, and strong stretchability. Notably, strong π–π stacking interactions between aromatic rings and multiple intermolecular hydrogen bonds also contribute to TC enhancement. When heated from room temperature to 120 °C, the polymer films exhibit stable morphology and uniform temperature distribution, indicating excellent thermal stability and efficient heat transfer properties (Fig. 4c). Notably, the well-balanced thermal, mechanical, and electrochemical properties of the polymer electrolyte significantly enhance the cycling stability of a Li–Li symmetric cell by effectively suppressing the growth of lithium dendrites.

Wang *et al.* developed a polyurethane-based single-ion conducting polymer electrolyte with sulfonamide side chains to enable fast Li^+^ transport (Fig. 4d).^115^ The hard domains, composed of aromatic rings, and the soft phase, consisting of flexible poly(ethylene oxide), provide high mechanical strength and elasticity. Improved Li^+^ conductive pathways are achieved through covalent tethering of anionic sulfonamide side chains to the hard segments and the solvent effect of the amorphous soft phases (Fig. 4e). The intense hard segments in the backbone and strong hydrogen bonding as physical cross-links reduce phonon scattering, thereby enhancing the polymer's TC.

The dual-phase concept has also been adopted in the design of dynamic supramolecular ion-conducting elastomers (DSICE) in our group.^116^ This design effectively addresses the trade-off between ionic conductivity, mechanical compatibility, and thermal properties. Furthermore, the introduction of dynamic disulfide bonding and stronger supramolecular quadruple hydrogen bonding in the hard phase endows the supramolecular structure with self-healing capability and favorable recyclability (Fig. 4f). These advancements in polymer electrolyte design highlight the potential for developing high-performance, thermally stable, and mechanically robust electrolytes for next-generation solid-state batteries.

#### Inorganic-polymer composite electrolyte

Inorganic-polymer composite electrolytes (IPCEs), which combine the advantages of inorganic fillers and organic polymers, are considered a promising approach to systematically improve ionic conductivity, mechanical properties, electrochemical stability, and thermal stability.^117,118^ While most studies have focused on enhancing these properties, limited attention has been given to the additional benefit of improved TC through the incorporation of inorganic fillers into the polymer matrix. Here, we discuss in detail how the intrinsic thermal properties of fillers, their arrangement, and the induced alignment of polymer molecules influence the TC of IPCEs.^119,120^

**Fig. 5:**
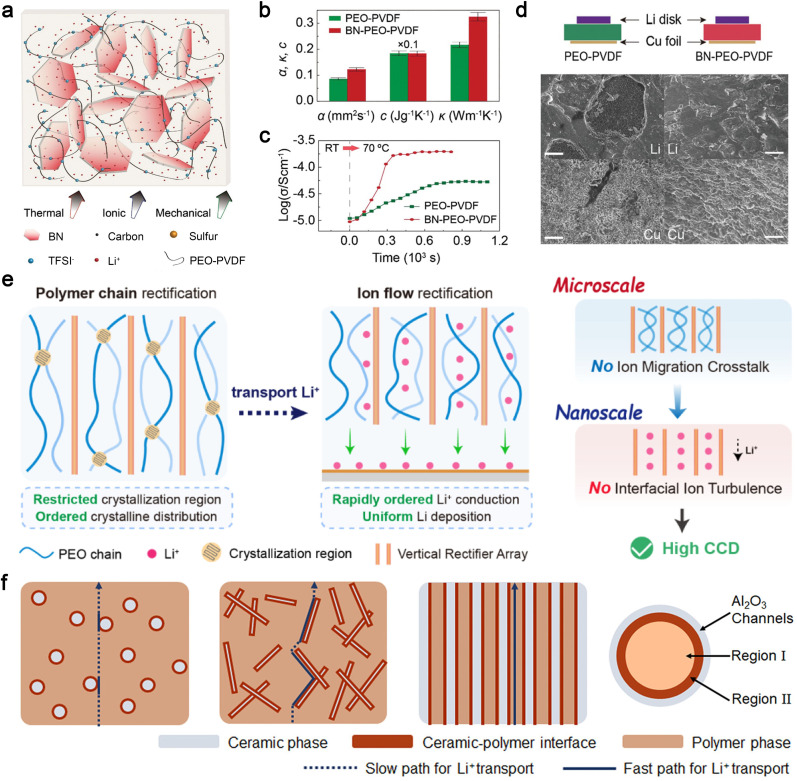
(a) Sketch of a composite electrolyte consisting of 2D BN flakes and a blended PEO-PVDF polymer with lithium bis(trifluoromethanesulfonyl)imide, (b) thermal diffusivity (*α*), heat capacity (*c*), and TC (*κ*) of PEO-PVDF and BN-PEO-PVDF electrolytes, (c) changes of ionic conductivities upon a temperature change, (d) sketch of Li–Cu cells and SEM images of Li metal on the surfaces of Li disks and Cu foils after Li deposition. Reproduced from ref. 121, copyright 2020, Wiley-VCH. (e) Schematic illustration of the critical current density enhancement of PEO electrolyte by a cross-scale rectification strategy. Reproduced from ref. 131, copyright 2024, Elsevier. (f) Schematics of composite solid polymer electrolytes with three types of geometrical structures of ceramic–polymer interface. Reproduced from ref. 137, copyright 2018, American Chemical Society.

Incorporating well-dispersed inorganic fillers with high intrinsic TC and electrochemical inertness into polymer electrolytes is a straightforward method to enhance heat transfer in composite electrolytes. For example, Zheng *et al.* developed a novel polyethylene oxide (PEO)-based electrolyte with improved thermal response by incorporating 2D BN nanoflakes (Fig. 5a).^121^ The BN additive was found to enhance ionic conductivity, mechanical strength, and heat transport in the PEO-based electrolyte. As shown in Fig. 5b, the thermal diffusivity (*α*) and TC (*κ*) of BN-PEO-PVDF electrolytes were significantly higher than those of PEO-PVDF electrolytes, owing to the high TC of BN.^122–124^ Accordingly, the enhanced thermal response of the BN-PEO-PVDF electrolyte facilitates faster heat equalization and more homogeneous ionic transport in the composite electrolyte (Fig. 5c). The electrochemical behavior of the Li anode with the BN-PEO-PVDF electrolyte was investigated using a Li–Cu asymmetric cell. After Li deposition, a uniform stripping pattern is observed on the Li surface, and the plated Li metal on the Cu foil also appears uniform. In contrast, significant pits are observed on the Li foil surface in the cell with the PEO-PVDF electrolyte, and the deposited Li on the Cu side exhibits unevenly distributed dense and porous regions (Fig. 5d). Furthermore, both the Li metal anode and S cathode demonstrate uniform and stable transformations during electrochemical reactions in an all-solid-state Li–S cell with the BN-PEO-PVDF electrolyte, resulting in superior performance characterized by high specific capacity, as well as good cyclic and rate behaviors.

The overall TC of organic polymer materials is influenced by factors such as molecular chain structure, degree of crystallinity, molecular chain orientation, and interchain interactions.^125,126^ While high crystallinity is detrimental to ionic conductivity, improved orientation of polymer molecular chains (from nano- to microscale) benefits both thermal and ionic conductivity in polymer electrolytes.^127–130^ Ding *et al.* developed a novel ion rectifier consisting of vertical arrays of copper-ion montmorillonite (Cu-MMT) and gelatin.^131^ This design offers two key advantages: first, the regular micron-sized vertical array structure achieves polymer chain rectification on the microscale by suppressing crystalline growth and distribution in non-ionic conduction directions, which also enhances TC along the vertical direction. Second, the anchored anions on the Cu-MMT surface and the abundant oxygen-containing groups in gelatin weaken the binding of Li^+^ by the PEO chain, enabling fast and uniform Li^+^ diffusion on the nanoscale (Fig. 5e).

The Cui group has extensively studied the effects of nanofillers on the ionic conductivity and electrochemical stability of polymer electrolytes.^132–136^ Their research demonstrated that the oriented alignment of nanowire fillers significantly enhances ionic conductivity by providing prolonged fast transport pathways for Li^+^, as Li^+^ conduction at the interfaces of nanowire crossing junctions is poor (Fig. 5f).^137^ Additionally, the improved polymer orientation within the confined space of aligned ceramic nanotemplates synergistically enhances the TC of composite polymers. In this approach, the polymer material is melted and infiltrated into a porous template, which orders the molecular chains as they flow into the nanoscale pores, thereby increasing TC. Furthermore, the high-TC ceramic nanotemplate (*e.g.*, Al_2_O_3_)^138^ provides additional TC pathways, achieving substantial heat dissipation enhancement through a “one stone, two birds” effect. These advancements highlight the potential of IPCEs to address critical challenges in battery performance by simultaneously improving thermal and ionic conductivity, mechanical strength, and electrochemical stability.

#### Control of polymer crystallinity domains

To address the challenge of simultaneously enhancing both ionic and TC in polymer electrolyte design, the confinement of crystallized polymer domains at the microscale and the construction of Li^+^ pathways at crystalline boundaries offer a novel approach.^139–141^ Wang *et al.* reported molecular ionic composite electrolytes based on an aligned liquid crystalline polymer combined with ionic liquids and concentrated Li salt, which exhibit high strength, non-flammability, high ionic conductivity, and electrochemical stability.^142^

**Fig. 6:**
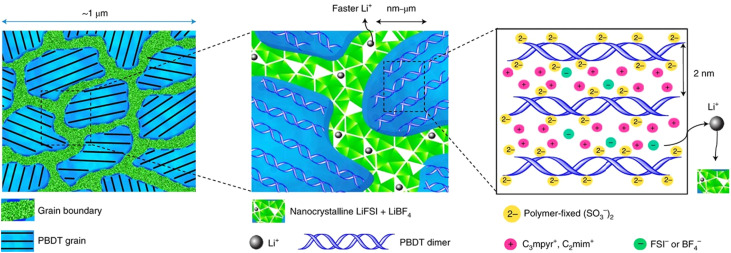
The microscale structure of a Li-loaded MIC. The grain boundaries are predominantly the condensed salt phase, which consists of nanocrystalline grains that form a conductive network supporting fast Li^+^ transport. The morphology of an aligned LC grain contains PBDT double helical rods filled predominantly with mobile ionic liquid cations. Reproduced from ref. 142, copyright 2021, Springer Nature.

Fig. 6 illustrates the micrometer-scale structure of this solid electrolyte, which incorporates aligned poly-2,2′-disulfonyl-4,4′-benzidine terephthalamide (Li-form PBDT) grains interleaved with a nanocrystalline ionic phase. These interconnected nanocrystalline grain boundaries separate individual PBDT grains, forming an additional conductive network that facilitates fast Li^+^ transport. Furthermore, the aligned liquid crystal (LC) grains containing PBDT double helical rigid rods enhance the thermal properties of the molecular ionic composite (MIC), providing high thermal stability and conductivity.^143^ This innovative design leverages the confinement of polymer crystallinity domains to optimize both ionic and thermal transport, offering a promising solution for developing high-performance polymer electrolytes.

#### Cathode materials

Lithium-rich oxides, known for their high energy density, are prone to decomposition above 200 °C, leading to oxygen release and exacerbation of exothermic reactions. To address this issue, previous studies have focused on improving the thermal stability of cathodes at high temperatures through methods such as coating treatments, elemental doping, anion acceptor addition, and structural engineering.^16,144^

From another perspective, cathode materials in LIBs typically exhibit higher TC than polymer-based separators and electrolytes.^145^ Enhancing heat dissipation efficiency at the cathode/separator and cathode/collector interfaces is crucial to prevent internal heat accumulation and material fatigue.^146^ He *et al.* conducted both theoretical and experimental research on methodologies to facilitate interfacial heat transfer across material components.^147^ They found that small organic molecules (SOMs) with unique functional groups (*e.g.*, –NH_2_) assembled at the cathode (LiCoO_2_, lithium cobalt oxide (LCO))-separator (PE) interface significantly enhance interfacial heat transfer. This improvement is attributed to the development of dual heat pathways through strong non-bonded interactions at the LCO-SOM and PE-SOM interfaces, high compatibility between SOMs and PE, and reduced phonon scattering.

#### Anode materials

Graphite, silicon, lithium metal, and alloy materials are commonly used as anode materials in LIBs.^148–150^ Since the vulnerable SEI on the anode surface is prone to decompose at elevated temperatures, leading to more violent redox reactions between the lithiated anode and the electrolyte, significant efforts have been made to improve the stability of the SEI through various organic and inorganic coating strategies. These strategies aim to reduce or delay the heat release from the anode.^151–153^

Improving TC within the anode, the anode/collector interface, and the anode/electrolyte interface can also effectively prevent SEI decomposition by directly lowering the temperature below the critical point.^154,155^ To this end, incorporating high-TC materials or enhancing thermal contact through high compression are facile and effective approaches that can be adopted.^156^ Notably, a recently reported strategy for improving the thermal stability of zinc electrodes by enhancing heat transfer in zinc-ion batteries can also be applied to lithium metal batteries, as both systems operate under similar mechanisms. In this work, thermal transfer-enhanced layers were coated on both sides of a zinc foil.^157^ The top layer, composed of zinc–alginate, polyacrylamide, and BN, enables a uniform Zn^2+^ flux and temperature distribution, while the bottom layer, consisting of Ag/Cu coating, improves local heat diffusion and mechanical stability. This dual thermal protection effectively suppresses thermodynamically driven dendrite growth and side reactions.

### External thermal regulation

External thermal management is critical for the safe operation of LIBs by directly dissipating excess heat generated during charge/discharge cycles.^158^ While numerous cooling technologies have been reported in recent years, this section focuses on recent advancements in the materials design of passive cooling, which offers notable advantages such as energy efficiency, compatibility, tunable functionality, and low maintenance cost. These include functional thermally conductive materials,^159^ PCMs,^160,161^ and radiative cooling materials.^162^

#### Functional thermally conductive materials

Thermally conductive materials play a vital role in battery thermal management by effectively propagating heat to regulate battery temperature and ensure operation within a safe range. Various thermally conductive materials with carbon or metal matrices have been developed, each with distinct advantages. Carbon-based composites, such as graphene,^163^ carbon nanotubes/nanofibers,^164,165^ graphite nanoplatelets,^166^ expanded graphite,^167^ and carbon aerogels,^168^ are attractive due to their high TC, light weight, structural designability, and multifunctionality. In contrast, metal-based composites (*e.g.*, metal foams,^169^ metal nanoparticles,^170^ and liquid metals (LMs)^171^) offer high TC, structural stability, and enhanced mechanical strength but face challenges such as heavy weight and corrosion risks. Further advancements in materials with high TC, lightweight properties, and reduced interfacial heat resistance are still needed.

**Fig. 7:**
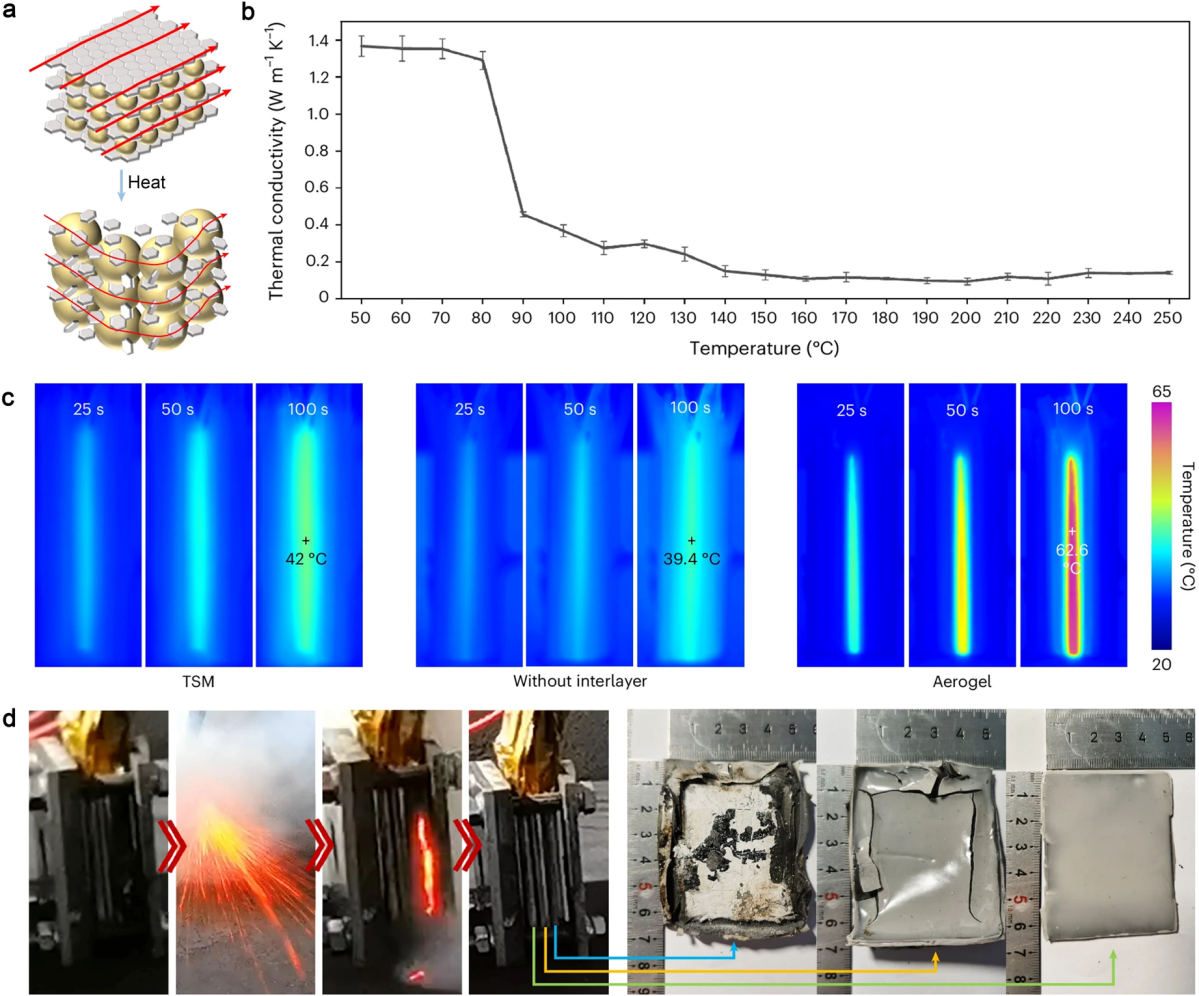
Schematic illustration of TSM design and thermal management test. (a) Thermal-switching mechanism of the TSM, (b) temperature-dependent TC of the TSM, (c) infrared images of battery modules with the TSM, with an aerogel and without an interlayer during the thermal dissipation tests, (d) the TSM remains functional as a smart thermal-protection layer throughout the thermal runaway propagation. The morphologies of the TSM change with the distance from the heat source. Reproduced from ref. 173, copyright 2024, Springer Nature.

In addition to the high-TC design principle, another strategy has emerged to manage both performance and safety of battery modules through rapid temperature-responsive thermal regulators.^172–174^ A thermal-switching material (TSM) composed of thermally expansive microspheres embedded between connected graphene layers has been designed, exhibiting a high switch ratio from thermal conduction to thermal insulation (Fig. 7a and b).^173^ Under normal operating conditions, the thermal regulator in its thermally conductive state (1.33 W m^−1^ K^−1^) buffers accumulated heat and reduces temperature variations between cells to less than 5 °C within 50 s, improving the electrochemical performance of the battery pack (Fig. 7c). When the temperature exceeds 100 °C, the TSM switches to a thermally insulating state to prevent thermal runaway propagation and battery explosions. It has been demonstrated that 80% of the total heat released during thermal runaway can be blocked by the responsive thermal-switching cell-to-cell TSM interlayer, successfully preventing uncontrolled chain reactions (Fig. 7d).

#### PCM composites

PCMs are widely applied in battery thermal management due to their ability to buffer drastic temperature changes by absorbing and releasing large amounts of transient heat, thereby maintaining the battery within a favorable temperature range. Among various PCMs composed of organic,^175^ inorganic,^176^ and eutectic compounds,^177^ organic PCMs are particularly advantageous due to their non-corrosive, non-toxic, and chemically stable nature. However, the low TC of organic PCMs not only limits cooling efficiency but also increases the risk of thermal runaway if heat builds up to the material's flash point. Encapsulating PCMs into conductive matrices to form phase changing composites (PCCs), including carbon-based,^178^ metal-based,^179^ ceramic-based,^180^ and multi-filler composites,^181^ has been identified as an effective solution to these challenges.

#### Carbon-based PCCs

Carbon-based composites, which combine the high latent heat of organic PCMs with the high TC, light weight, and chemical stability of carbon materials, are particularly attractive for battery thermal management.^182^ To date, the most commonly reported carbon matrices include carbon fibers (CFs),^183^ carbon nanotubes (CNTs),^184^ expanded graphite (EG),^185^ graphene,^186^ and others.

**Fig. 8:**
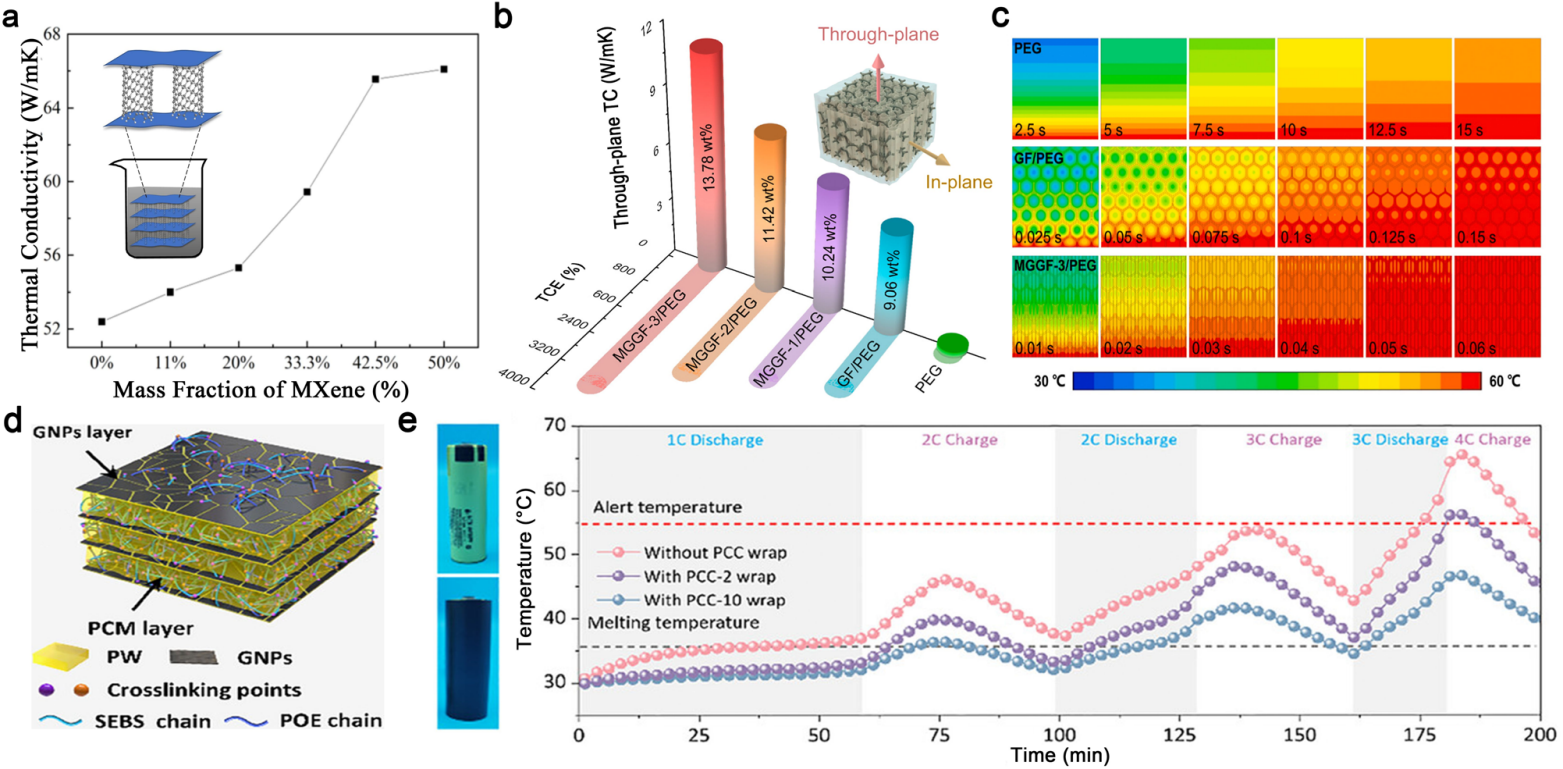
Carbon-based PCCs. (a) TC of the CNT@MXene aerogel with different mass ratios. (a) Reproduced with permission.^191^ copyright 2025, Elsevier. (b) Through-plane TC of PEG, GF/PEG, and MGGF/PEG, (c) simulated transient heat flux distribution. Reproduced from ref. 192, copyright 2023, Elsevier. (d) Phase change composite (PCC) with highly oriented layered structures, (e) surface temperature evolution of a battery with and without a PCC wrap during continuous charging and discharging. Reproduced from ref. 193, copyright 2025, Wiley-VCH.

One-dimensional carbon fillers are well-suited for LIB thermal management due to their high axial TC, high strength, and high temperature resistance.^187,188^ Unlike disordered CNT arrangements, aligned CNTs leverage the intrinsic high axial TC of CNTs and provide continuous heat conduction pathways, significantly enhancing the thermal properties of CNT/PCM composites.^189^ Additionally, aligned CNTs exhibit higher paraffin loading capacity and significantly increase latent heat storage density.^190^ CNTs can also be combined with other 2D thermally conductive materials to create continuous pathways for heat transfer in PCCs. For example, Lin *et al.* introduced a novel CNT/MXene aerogel with a 3D porous structure for PCC preparation.^191^ The interconnected CNT pillars and MXene nanosheets, held together by hydrogen bonding and electronegativity, provide thermal transfer pathways in both horizontal and vertical directions, resulting in significant TC improvement (Fig. 8a). In battery thermal management, this composite demonstrates rapid improvement in temperature distribution, reducing the temperature rise rate from 1.85 °C to 0.92 °C under extreme high-rate (4C) charging/discharging conditions.

EG is widely used as a conductive skeleton due to its accessibility and low cost. Its hierarchical pores of micro-, meso-, and macro-sizes are particularly useful for encapsulating liquid PCMs, which are prone to leakage.^194^ EG/PCM composites with high latent heat and TC can effectively dissipate heat within a short time and prevent the propagation of thermal runaway processes.^195,196^ Notably, graphene, a well-known 2D material with high in-plane TC, can be constructed into 3D thermally conductive networks through strong intermolecular interactions or chemical bonding, further enhancing the TC of PCCs.^197–199^ Li *et al.* developed a PEG composite based on a dual-encapsulation design of vertically aligned MXene-graphene monoliths in graphene foam (MGGF).^192^ Impressively, the resultant MGGF/PEG composite achieved a high through-plane thermal conductivity of 11.39 W m^−1^ K^−1^ and a desirable latent heat density of up to 160.3 J g^−1^ (Fig. 8b). From the simulated transient heat flux distribution, it was shown that the heat flow is primarily distributed along the MGGF skeleton, which can notably promote the heat transfer rate by 2.5 times and 416.7 times compared to graphene foam (GF)/PEG and pure PEG, respectively (Fig. 8c).

Recently, incorporating functional polymers into PCCs has been shown to offer multiple advantages, such as reducing interfacial thermal resistance by enhancing contact between the composite PCMs and batteries, improving leak-proof properties through physical coating or chemical cross-linking, and providing high latent heat due to the use of multi-PCMs.^11,200,201^ Hu *et al.* designed a novel PCC using styrene–butadiene–styrene (SBS) thermoplastic elastomer as the polymer framework and CFs as thermally conductive fillers.^202^ The SBS framework provides intensive capillary condensation to adsorb the liquid-state paraffin PCM, thereby improving leak-proof properties. At an ambient temperature of 40 °C, the composite controlled the maximum temperature and temperature difference of the battery module to below 49.23 °C and 4.76 °C, respectively, at a high charge–discharge rate of 3C. These values are 9.71 °C and 1.89 °C lower than those achieved with natural air cooling. Moreover, a flexible, highly conductive, and recyclable PCC has been developed by employing a dual-polymer network of styrene–ethylene–butylene–styrene (SEBS) and polyolefin elastomers (POE), along with the integration of shear-induced alignment of graphene nanoplatelets (GNPs), as shown in Fig. 8d.^193^ The dynamic physical crosslinking network of SEBS and POE endows the PCCs with robust structural stability, tunable flexibility, and heat induced self-healing functionality. In the thermal management demonstration, the PCC-wrapped battery exhibits much lower temperature than the bare battery, particularly during high-rate charging and discharging (Fig. 8e), suggesting practical opportunities for preventing overheating and reducing the risk of thermal runaway in batteries, especially in fast charging and discharging processes.

#### Metal-based PCCs

Porous metallic conductive networks, composed of metal fibers,^203^ metal mesh,^204^ and metal foam,^205^ provide metal-based PCCs with excellent TC and mechanical strength. Both the morphology of metallic fillers and the geometric structure of metal foam significantly influence the thermal dissipation properties of PCCs.

Cheong *et al.* reported a vertically structured metal foam with superior heat diffusion performance compared to five other metal foam structures.^206^ The vertical structure achieves a faster melting rate of PCMs and uniform temperature distribution, and reduces the maximum temperature by approximately 96.8 °C. Wang *et al.* innovatively developed bionic antler-like microtopological copper fibers and applied them as a porous metal matrix to enhance phase change heat transfer.^207^ Experimental results demonstrated that the copper fiber sintered mat matrix enhances the internal heat transport efficiency in PCMs, with particularly significant TC improvement under high heat flux conditions. Additionally, studies have shown that gradient porosity and localized foam filling can further improve the heat transfer rate in PCMs, which should be considered in the design of porous metal-based PCCs.^208^

Recently, LMs have emerged as promising PCMs for thermal management in electronics and batteries due to their high TC and latent heat per unit volume.^209^ A scalable micro-encapsulated PCM and eutectic gallium–indium LM integrated composite demonstrated superior heat mitigation compared to pin-fin heat sinks, increasing full-load operation time by 4.14 times.^210^ This composite also shows potential for regulating transient temperature rises in fast-charging batteries under high heat flux conditions. However, gallium LM may suffer from severe supercooling, where molten gallium does not solidify due to the lack of nucleation sites. Ki *et al.* proposed a strategy of infusing gallium into porous copper to form intermetallic compound impurities at the interfaces, reducing the activation energy for heterogeneous nucleation.^160^ During repetitive heating–cooling cycles, porous-shaped gallium consistently exhibits crystallization propagation near room temperature while maintaining stable performance as a thermal buffer, making it suitable for mitigating rapid temperature increases in batteries.

#### Ceramic-based PCCs

Ceramics, such as BN and aluminum nitride, are ideal fillers for PCCs in battery thermal management due to their high TC, electrical insulation properties, and thermal stability.^211,212^

**Fig. 9:**
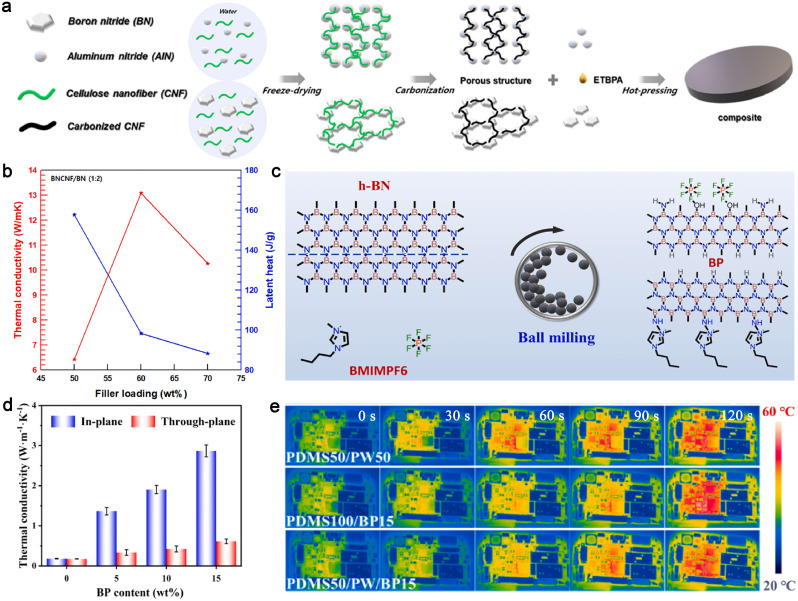
Ceramic-based PCCs. (a) Fabrication process of ETBPA composites, (b) TC and latent heat of the ETBPA/BNCNF/BN composites. Reproduced from ref. 213, copyright 2023, Elsevier. (c) Preparation process diagram of BPs, (d) *K*‖ and *K*⊥ of PDMS50/PW50/BP, (e) infrared thermal imaging picture of different composites for thermal management of mobile phones. Reproduced from ref. 214, copyright 2024, Elsevier.

Lee *et al.* developed a highly thermally conductive PCC using a porous ceramic skeleton of CF-crosslinked BN and a novel PCM derived from erythritol and bisphenol A grafting (denoted as ETBPA) (Fig. 9a).^213^ The results demonstrate that the in-plane TC of the composite exceeds 13.09 W m^−1^ K^−1^, which is more than 25 times higher than that of pure ETBPA, while maintaining a high latent heat of 98.4 J g^−1^ (Fig. 9b).

Additionally, the oriented alignment of BN can significantly enhance directional thermal transfer efficiency. To achieve this, Huang *et al.* developed a scraping coating technology to construct highly oriented BN nanosheets in a PDMS/PW matrix using strong shearing forces along the blade-casting direction.^214^ BN nanosheets modified with 1-butyl-3-methylimidazolium hexafluorophosphate (BMIMPF_6_) ionic liquid (denoted as BPs) were first prepared from bulk BN powders and BMIMPF_6_ ionic liquid through a one-step ball milling process (Fig. 9c). The resulting composites exhibited a high in-plane TC of 2.87 W m^−1^ K^−1^ due to the interconnected BN thermally conductive network (Fig. 9d). The high TC of the aligned BN network, combined with the thermal energy buffering of PW, enabled effective heat dissipation, reducing the working temperature of smartphones by over 11 °C (Fig. 9e).

#### Radiative cooling materials

Radiative cooling technology provides a low-energy and sustainable solution to address cooling challenges. The principle of radiative cooling is to emit the thermal energy of objects into outer space in the form of electromagnetic waves through an atmospheric transparent window (typically within the wavelength range of 8–14 µm). High-performance radiative cooling can be achieved by utilizing materials with high intrinsic emissivity in the atmospheric window (*e.g.*, SiO_2_, Al_2_O_3_, graphene)^215–218^ and employing advanced structural designs such as photonic crystals,^219^ metamaterials,^220^ and porous structures.^221^

**Fig. 10:**
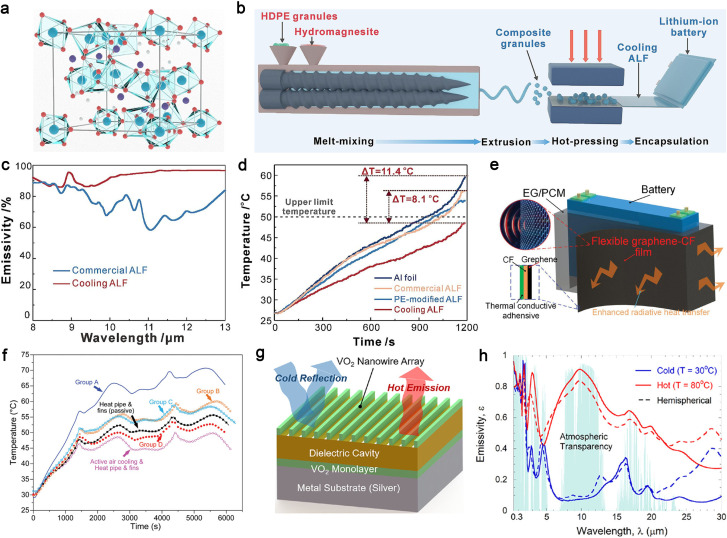
Thermal radiation materials. (a) Crystal structure of hydromagnesite in a polyhedral form. The Mg, O, C and H atoms are blue, red, purple, and white, respectively, (b) schematic diagram of the preparation process for radiative cooling ALF, (c) emissivity spectra of commercial and cooling ALFs, (d) comparison of temperature rise of LIBs encapsulated in different ALF samples at a 3C discharging rate. Reproduced from ref. 222, copyright 2025, Elsevier. (e) Schematics of the EG/PCM/graphene composite for passive battery thermal management, (f) thermal comparison among the various battery thermal management methods. Group D represents the EG/PCM/graphene composite, while Groups A, B, and C are control samples. Reproduced from ref. 223, copyright 2024, Wiley-VCH. (g) Illustration and schematic of VO_2_ nanowire/dielectric material/VO_2_ sub-monolayer coating on a Ag substrate, (h) the averaged emissivity spectrum of the optimized VO_2_ nanowire/BaF_2_/VO_2_ on Ag coating calculated with rigorous coupled-wave analysis. Reproduced from ref. 224, copyright 2022, Elsevier.

Chen *et al.* reported an industry-scalable radiative cooling technology using hydromagnesite-based composites with excellent selective optical responses.^222^ Hydromagnesite (Mg_5_(CO_3_)_4_(OH)_2_·4H_2_O, Fig. 10a) has a crystal structure consisting of octahedral MgO_6_ centers and carbonate ions, both of which exhibit multiple mid-infrared vibrations. The manufacturing process for radiative cooling aluminum laminated foil (ALF), the commercially used outer packaging of pouch LIBs, involves a simple two-step process. Hydromagnesite particles and a high-density polyethylene (HDPE) matrix are melt-blended to form homogeneous compound particles, which are then hot-pressed onto aluminum foil to encapsulate the battery (Fig. 10b). The resulting radiative cooling ALF exhibits much higher emissivity than commercial ALF (Fig. 10c), enabling efficient thermal management of LIBs by regulating battery temperature within an optimal operating range at a high 3C discharge rate (Fig. 10d).

However, addressing cooling demands with individual cooling technologies remains challenging in scenarios with substantially higher heat fluxes. To address this, Mao *et al.* proposed an integrated thermal management technology using an EG/PCM/graphene composite.^223^ The EG/PCM acts as a composite PCM with effective TC and latent heat, tightly wrapped around a battery pack. Nanostructured graphene is coated on the composite PCM with a copper foil in between to ensure efficient heat transfer. The graphene outer surface functions as a thermal radiator, efficiently dissipating the heat generated within the composite PCM (Fig. 10e). In battery cooling experiments at a 2.50C charge/discharge rate and 30 °C ambient temperature, the EG/PCM/graphene composite outperformed control samples with single cooling mechanisms (heat storage *via* phase change or radiative cooling). Furthermore, it demonstrated superior cooling performance compared to passive heat pipe approaches and was comparable to active air cooling and heat pipe schemes (Fig. 10f).

More recently, switchable radiative thermal management, which enables batteries to operate at optimal temperatures using emissivity-changeable materials under high and low temperatures, has gained attention for its adaptability to different application scenarios. Zhang *et al.* proposed a monolithic high-performance turn-down thermal emittance coating, consisting of a VO_2_ sub-wavelength nanowire grating top layer, an index-matched Fabry–Perot dielectric thin film middle layer, and an additional absorbing VO_2_ sublayer (Fig. 10g).^224^ Leveraging the insulator-to-metal temperature phase transition of VO_2_, this coating enables responsive passive radiative cooling through emissivity switching when the temperature exceeds the transition threshold (Fig. 10h). This innovation holds promising potential for self-cooling of batteries at high temperatures.

## Conclusions and outlook

### Conclusions

Based on a detailed analysis of the thermal runaway mechanisms in LIBs, it can be concluded that heat accumulation within LIBs leads to thermal runaway through a long-term process of chain (or crosstalk) reactions or dendrite growth-induced short circuits. Establishing efficient heat dissipation pathways both inside and outside the battery is crucial to mitigate or even prevent thermal runaway. Internal heat dissipation plays an irreplaceable role in achieving a mild and uniform temperature distribution within the cells, primarily relying on the rational design of highly thermally conductive materials without compromising electrochemical performance. Meanwhile, external thermal management is indispensable, and material-based passive cooling technologies provide a continuous, efficient, and cost-effective solution for overheating batteries. With a clear understanding of heat transfer mechanisms guiding the design of organic, inorganic, and composite materials, this review emphasizes improving the intrinsic TC of polymers through molecular structure and orientation engineering, as well as synergistically enhancing TC in composites through highly efficient thermal conduction networks. Additionally, recent advancements in multifunctional materials featuring switchable thermal and ionic conductivity, high thermal storage capacity, and responsive radiative cooling have opened new avenues for research in intelligent thermal management technologies.

### Outlook

The ever-growing demand for high-density and fast-charging LIBs has imposed higher requirements for safe battery materials. However, reconciling the electrochemical and thermal performance of key materials remains a significant challenge. Unfortunately, electrochemical performance has often been prioritized in application-driven material design. Therefore, more systematic studies are required to address thermal issues without compromising electrochemical properties.

(1) Although solid-state LIBs are regarded as the next generation of high-density, safe, and fast-charging batteries, heat accumulation within the battery poses considerable safety risks, particularly at high charge/discharge rates, and should not be neglected. In polymer-based solid electrolytes, ionic conductivity is facilitated by flexible chain segments and polar groups, whereas phonon transport relies on rigid chain segments and multiple intermolecular hydrogen bonds. However, stronger intermolecular interactions can significantly restrict the motion of polymer chains, resulting in reduced ionic conductivity. This introduces a trade-off when integrating both soft and hard phases into the polymer structures. Decoupling the thermal and electrochemical properties of polymers through structural design remains a significant challenge.

(2) Organic–inorganic composite electrolytes, which combine the advantages of organic polymers and inorganic fillers, are regarded as the most promising solid electrolytes for high-performance solid-state LIBs. From a heat dissipation perspective, these composite electrolytes provide a versatile approach to synergistically enhance both thermal and ionic conductivity. Aligned inorganic templates with high thermal conductivity (*e.g.*, BN, Al_2_O_3_) not only facilitate heat dissipation within the composites but also induce molecular orientation upon infiltration, thereby simultaneously improving thermal and ionic conductivity. This holds promising potential for enhancing both safety and electrochemical performance. Further mechanistic investigations into the effects of polymer/inorganic interfacial interactions on phonon scattering, ionic dissociation, and transport behavior are crucial to advancing the development of these materials.

(3) Heat accumulation can be effectively mitigated through the design of highly thermally conductive materials for both internal cell components and external thermal management systems. However, in cases of external abuse triggering instantaneous release of enormous amounts of heat during thermal runaway, existing heat dissipation mechanisms may fail to cool the overheated battery, posing a high risk of fire or explosion. Under such extreme abuse conditions, integrating switchable shutdown functions, derived from the temperature-dependent reversible expansion and contraction of polymer materials, into thermally conductive components within or between cells provides a feasible and rapid response mechanism to mitigate thermal runaway risks.

(4) This review primarily focuses on preventing thermal runaway by dissipating excess heat from the cell and maintaining a homogeneous temperature distribution under optimal working conditions. As a key aspect of external thermal management technologies, composite PCMs with high TC play a dual role: storing heat in cooling mode at high temperatures and feeding heat back at low temperatures when batteries are used in cold regions during winter. This multifunctional thermal management approach can be further enhanced by incorporating passive radiative cooling technology utilizing a VO_2_ metasurface with tunable thermal emittance, providing a smart, continuous, energy-efficient, and versatile thermal management solution for LIBs.

## Author contributions

Songpei Nan: writing – original draft preparation, visualization, review & editing. Guoxin Gao & Wei Yu: visualization, review & editing, funding acquisition. Shujiang Ding: supervision, review & editing, funding acquisition. Dawei Ding: writing – original draft preparation, supervision, review & editing, funding acquisition.

## Conflicts of interest

There are no conflicts to declare.

## Data Availability

No primary research results, software or code have been included and no new data were generated or analysed as part of this review.
